# The Plant Ontology Facilitates Comparisons of Plant Development Stages Across Species

**DOI:** 10.3389/fpls.2019.00631

**Published:** 2019-06-04

**Authors:** Ramona L. Walls, Laurel Cooper, Justin Elser, Maria Alejandra Gandolfo, Christopher J. Mungall, Barry Smith, Dennis W. Stevenson, Pankaj Jaiswal

**Affiliations:** ^1^CyVerse, Bio5 Institute, The University of Arizona, Tucson, AZ, United States; ^2^Department of Botany and Plant Pathology, Oregon State University, Corvallis, OR, United States; ^3^Liberty Hyde Bailey Hortorium, Plant Biology Section, School of Integrative Plant Science, Cornell University, Ithaca, NY, United States; ^4^Environmental Genomics and Systems Biology, Lawrence Berkeley National Laboratory, Berkeley, CA, United States; ^5^Department of Philosophy, University at Buffalo, Buffalo, NY, United States; ^6^New York Botanical Garden, Bronx, NY, United States

**Keywords:** Arabidopsis, BBCH, Gene Ontology, ontology, plant development, plant embryo, *Zea mays*

## Abstract

The Plant Ontology (PO) is a community resource consisting of standardized terms, definitions, and logical relations describing plant structures and development stages, augmented by a large database of annotations from genomic and phenomic studies. This paper describes the structure of the ontology and the design principles we used in constructing PO terms for plant development stages. It also provides details of the methodology and rationale behind our revision and expansion of the PO to cover development stages for all plants, particularly the land plants (bryophytes through angiosperms). As a case study to illustrate the general approach, we examine variation in gene expression across embryo development stages in Arabidopsis and maize, demonstrating how the PO can be used to compare patterns of expression across stages and in developmentally different species. Although many genes appear to be active throughout embryo development, we identified a small set of uniquely expressed genes for each stage of embryo development and also between the two species. Evaluating the different sets of genes expressed during embryo development in Arabidopsis or maize may inform future studies of the divergent developmental pathways observed in monocotyledonous versus dicotyledonous species. The PO and its annotation database (http://www.planteome.org) make plant data for any species more discoverable and accessible through common formats, thus providing support for applications in plant pathology, image analysis, and comparative development and evolution.

## Introduction

Studies that examine patterns of gene expression across species or environments are more powerful when they involve consideration of specific development stages, because they can control for variation in expression through ontogeny (e.g., Richards et al., 2012). However, comparing development across species is challenging, because biologists often use species- or clade-specific terminology to describe development stages. The Plant Ontology (PO, Walls et al., 2012a; Cooper et al., 2013) was designed as a species-neutral vocabulary for plant anatomy, morphology, and development stages that can be used to associate data to a common set of species-neutral terms for plant sciences. The PO provides not only nomenclature and definitions, but also relationships among terms that can be used for computerized logical inference (Smith et al., 2007; Washington and Lewis, 2008; Walls et al., 2012a). PO terms for development stages allow researchers to accurately describe at what stage during the life of a plant, or plant part, a specimen was collected, a physiological parameter was measured, or a gene was expressed. Shared terminology makes it easier to compare results from different species and experiments, or to identify developmental timing that differs from the norm within a species. Specifying the shared terminology in a machine-actionable, semantic format permits automated integration and analysis of much larger datasets than would otherwise be possible, allows new facts to be inferred from existing data, and supports discovery across multiple platforms. Development stage data are stored in the Planteome database^1^ as annotations that capture associations between a PO term and an entity such as a gene, gene model, protein, mutant phenotype, or Quantitative Trait Locus (QTL).

This communication first describes the methodology and rationale behind the revision and expansion of the PO to cover development stages for all plants, particularly the land plants (bryophytes through angiosperms). We then discuss how the PO facilitates comparison of data sets across taxa through the associations to plant genomic data made by tagging these data with terms from the PO. We provide an example comparison of gene function and expression in embryo development stages of Arabidopsis and maize, using data from the Planteome annotation database.

### Background

The PO is divided into two major branches rooted in the terms *plant anatomical entity*^2^ and *plant structure development stage*. The former branch was described in detail in Cooper et al. (2013). We focus here on the *plant structure development stage* branch and associated data. Historically, the growth and development of whole plants was described using scales that were specific to one species, or to a set of closely related species – for example the growth stages for members of the Tribe Triticeae as described in Zadok et al. (1974). An effort to unify whole plant stage descriptions across multiple crop species resulted in the BBCH scale (from the Biologische Bundesanstalt, Bundessortenamt und chemische Industrie) (Hack et al., 1992; Meier, 2001). Later work by Boyes et al. (2001) provided standardized growth stage nomenclature for the non-crop species *Arabidopsis thaliana* (Arabidopsis) based on the BBCH scale. The BBCH scale and its many extensions are still widely used across both annuals and perennials (e.g., Salinero et al., 2009; Archontoulis et al., 2010), but their descriptions are designed for agronomic practices that ignore finer details of ontogeny and comparative plant anatomy. This makes the BBCH scale inadequate for annotating and querying the volumes of detailed genomic and phenotypic data now available to plant scientists. This need prompted several plant database organizations to develop whole plant growth stage vocabularies for particular taxa (Garcia-Hernandez et al., 2002; Rhee et al., 2003; Vincent et al., 2003; Yamazaki and Jaiswal, 2005). In 2006, separate vocabularies for Arabidopsis, *Zea mays* (maize), and *Oryza sativa* (rice) were merged into a unified ontology, known as the Whole-Plant Growth Stage Ontology (GSO) (Pujar et al., 2006). Eventually, the GSO was merged with the Plant Structure Ontology to form the PO (Cooper et al., 2013).

Just as the BBCH’s focus on crop species was a serious limitation to its use with plants such as Arabidopsis, so the PO’s original focus on annual angiosperms limited its applicability to the new species for which genomic and phenomic data were becoming available. This led the developers of the PO to turn their attentions to non-angiosperm model species such as moss (*Physcomitrella patens*; Rensing et al., 2008) and spikemoss (*Selaginella moelendorfii*; Banks et al., 2011), perennial woody angiosperms such as poplar (*Populus trichocarpa*; Tuskan et al., 2006) and grape (*Vitis vinifera*; Jaillon et al., 2007), and tropical crop species such as banana (*Musa acuminate*; D’Hont et al., 2012). The results prompted the revision of many of the original PO representations of *plant structure development stages*, which had included only stages in the sporophyte phase. Although the sporophyte phase is the most relevant phase for gymnosperms and angiosperms, it is inadequate for describing bryophyte, lycophyte, and pteridophyte life cycles with prominent gametophyte stages.

## Materials and Methods

### Defining Development Stages in the PO

Terms for development stages in the PO may apply to all plants or only specific taxa, depending on the taxonomic distribution of the structure that is developing. For example, *sporophyte development stage* is defined in such a way that it describes the sporophyte phase (2*n* number of chromosomes) of bryophytes, lycophytes, pteridophytes, and seed plants (gymnosperms and angiosperms), whereas *whole plant flower development stage* is defined to describe only angiosperms, and *protonema whole plant development stage* only bryophytes and pteridophytes.

The PO terms are defined using the Aristotelian or *genus-differentia* format: An *A* is a *B* which *C*s (Rosse and Mejino, 2003; Arp et al., 2015). Here the genus term *B* represents the more general group of entities of which the term of interest *A* represents a subgroup. The differentiae *C* are those characteristics that set apart the members of this more specific subgroup *A* from the wider group *B*. Examples of the differentiae used to define *plant structure development stages* include:

• A detectable landmark such as the presence of a *plant structure*, e.g., the formation of a *vascular leaf primordium* defines the beginning of the *vascular leaf primordium formation stage.* Some landmarks are visible, while others are only detectable with molecular techniques.
• Occurrence of a *developmental process* from the Gene Ontology (GO), e.g., the *sporophyte senescent stage* is defined as *sporophyte development stage* during which a plant is undergoing GO: *multicellular organism senescence*.
• Involvement of a specific *plant structure*, e.g., a *plant tissue development stage* is defined as a *plant structure development stage* that has as a primary participant some *portion of plant tissue*.

Definitions are stored in two forms: as text, understandable to humans reading the ontology, and as logical axioms stored in the machine-readable language known as OWL (from the Web Ontology Language, Hitzler et al., 2012). Using the *genus-differentia* formula not only provides unambiguous definitions for terms; when combined with machine reasoners, it also supports automatic data classification (e.g., any plant structure that is undergoing a senescence process can automatically be associated with the *sporophyte senescent stage*).

### Relations Used in the PO

All relations in the PO come from the OBO (Open Biological and Biomedical Ontology) Relations Ontology (RO; Smith et al., 2005), except *subClassOf* (also known as *is_a*), which is part of the RDF Schema (Rdf Working Group, 2014). The latter is used to define a primary hierarchy of PO development stages, based on relations such as:

plant embryo development stage subClassOf sporophyte vegetative stage

**FIGURE 1:**
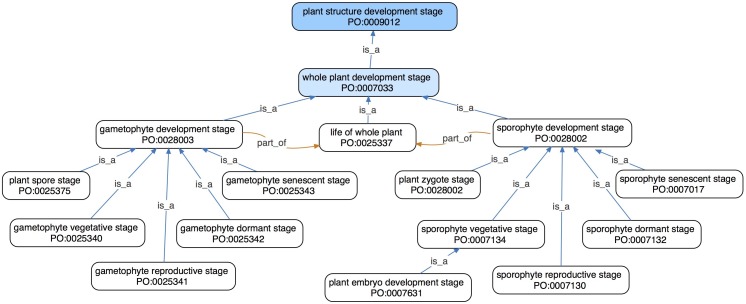
An ontology graph showing direct and second-level subclasses (blue arrows) in the *whole plant development stage* branch in the PO. Both the *sporophyte development stage* and *gametophyte development stage* have subclasses describing vegetative, reproductive, dormant, and senescent stages. Every instance of *sporophyte development stage* and *gametophyte development stage* is a part of (orange arrows) some *life of whole plant*, thus making *life of whole plant* a major hub/node of this graph.

**FIGURE 2:**
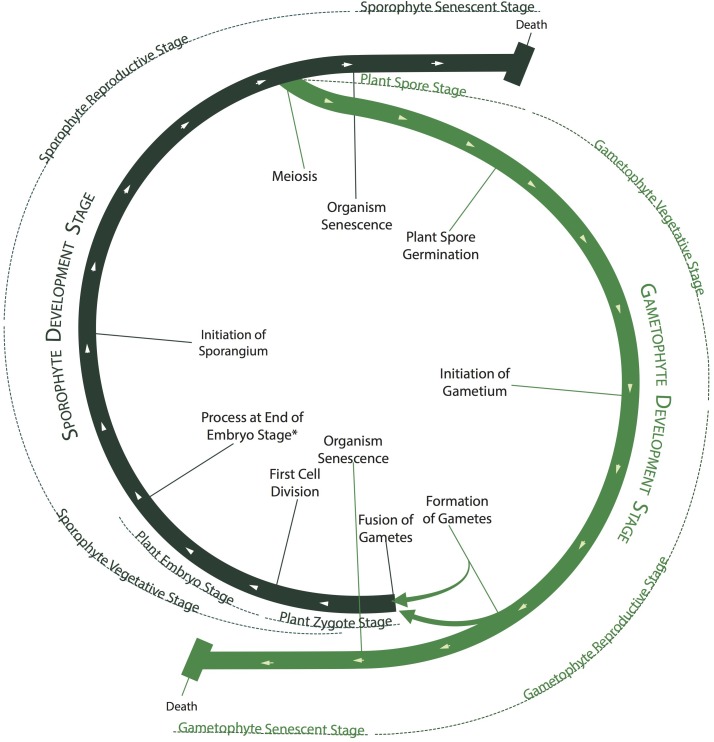
Green plant life cycle as represented by the PO. The developmental stages of plants are often arranged into a life “cycle” (thick green lines) in which the gametophyte and sporophyte phases alternate, although no individual plant undergoes the entire cycle. Different *plant structure development stages* in the PO occur sequentially along the cycle, as mapped by the thinner, external lines, although the senescent stages can begin at any point. The beginnings and endings of different development stages are defined by different developmental processes, shown as radial lines intersecting the thick green lines.

In this hierarchy, sub-stages in the development of a specific structure such as *whole plant*, *flower*, or *vascular leaf* correspond to time intervals in the growth of the corresponding *plant structure*, with shorter stages as subclasses of longer stages. For the example above, this means that every embryo stage occurs during a sub-interval of the time during which some sporophyte stage occurs (Figure 1, 2). This approach differs from that adopted in some development stage ontologies created for animal model organisms (e.g., Mungall et al., 2012), which build their hierarchies of more and less specific stages using the *part_of* relation (e.g., *blastula stage part of some cleavage stage* in Mungall et al., 2012). We chose to maintain the subclass of hierarchy used in earlier versions of the PO, as well as its predecessor the GSO, in order to maintain existing data annotations. This choice also allows us to use the *part_of* relations to link a developmental stage of one structure to the developmental stage of another structure of which it is a part, as in

floral organ formation stage part_of flower development stage

providing greater inferencing power.

*Plant structure development stages* may be related to *plant anatomical entities* using *has_participant* or *participates_in* relations. For example, the assertion:

plant spore development stage has_participant plant spore

means that the main participant or actor is a *plant spore*, even though other structures (such as a *sporangium*) also may be involved in that stage. The *participates_in* relation is used to link a *plant structure* to the *plant structure development stage* during which it must exist. For example,

protonema participates_in gametophyte development stage

should be read, in accordance with the RO every/some rule (Smith et al., 2005), to mean that every *protonema* exists during some *gametophyte development stage*. These relations are used in the PO primarily to enable data known to be associated with a stage or structure to be inferred as associated with the corresponding structure or stage.

**FIGURE 3:**
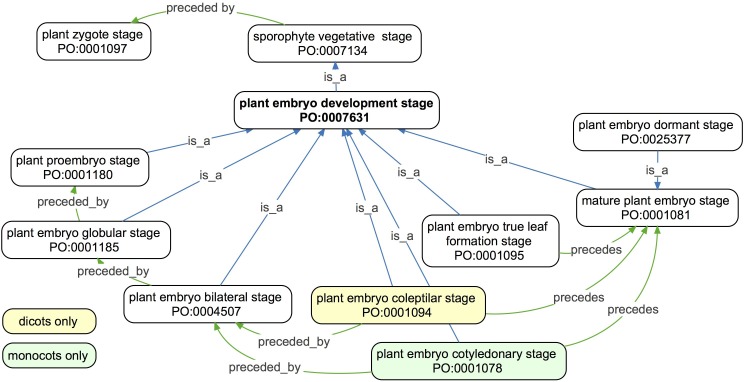
An ontology graph of the *Plant embryo development stage* and related terms in the PO. Developmental stages are ordered temporally using the *precedes* and *preceded by* relations. Because a *sporophyte vegetative stage* begins after the first division of the *plant zygote* in the *plant zygote stage*, all *plant embryo development stages* are preceded by a *plant zygote stage*. Therefore, *plant embryo development stage* is a subclass of *sporophyte vegetative stage*. The majority of the embryo development stages are defined commonly among taxa, but differences are highlighted in yellow (dicots only) and green colored boxes (monocots only).

The *precedes* and *preceded_by* relations are used to indicate that the end of one stage happens at or before the start of another stage. For example,

*plant proembryo stage* is *preceded_by plant embryo globular stage*

(Figure 3). The *preceded_by* relation is transitive, and thus provides a temporal order. This temporal order is used, for example, when we infer that *plant embryo bilateral stage* is *preceded_by* a *plant proembryo stage*, via the *plant embryo globular stage* (Figure 3). Progressively finer resolutions made possible by future developmental biology research studies may disclose intermediary stages not yet acknowledged. Many branches in the PSDS do not have ‘precedes’ or ‘preceded_by‘ relations for temporal ordering. Earlier developers of the PO used letters of the alphabet in order to arrange the subclass terms in temporal order on the browser display, for example the subclasses of *pollen development stage* and *megagametophyte development stage*. In other cases, the early developers used a letter and number code to order the subclasses, such as the subclasses of the *whole plant flowering stage*, which are named like *FL.00 first flower(s) open stage*. Work is ongoing to expand coverage of the *precedes* and *preceded_by* relations.

A more detailed description of all the relations used in the PO can be found online^3^.

### Associating Data With PO Terms

Information on how PO annotations are created is included in Supplementary Document S1 and discussed in Cooper et al. (2013) and in Cooper et al. (2018).

### Comparison of Gene Expression Across Stages and Species

**FIGURE 4:**
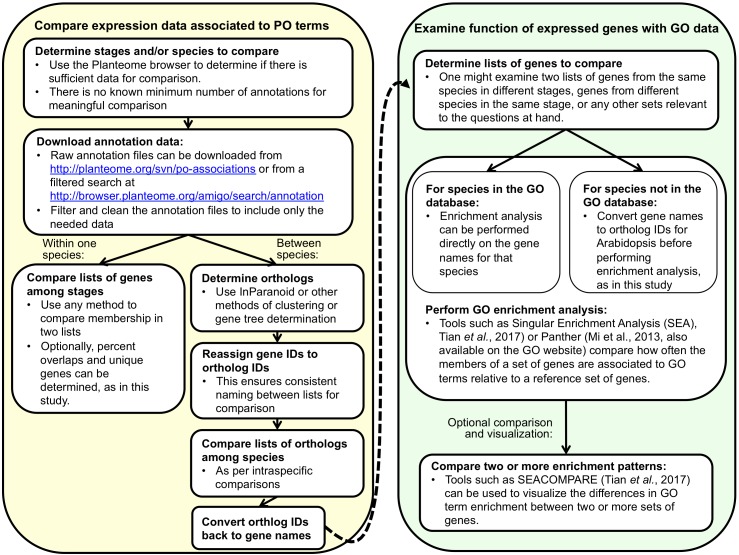
A generalized representation of the two-part workflow used in this study to compare gene expression between development stages within one species or between species within one stage. Alternative tools for many of the steps exist. Before undertaking such an analysis, it is important to consider the amount and quality of annotation data available for comparison, both in the PO and GO.

A generalized diagram of our workflow is shown in Figure 4. We used data annotated to the subclasses of *plant embryo development stage* to examine variation in gene expression across development stages and species. We downloaded annotation data from the PO subversion repository revision 412^4^ (filenames: po_temporal_gene_arabidopsis_tair.assoc and po_growth_genemodel_zea_maizeGDB.assoc) for the following stages: *plant proembryo stage, plant embryo globular stage, plant embryo bilateral stage, plant embryo coleoptilar stage, plant embryo cotyledonary stage*, *mature plant embryo stage*, and *plant embryo true leaf formation stage*. All annotations came from either Arabidopsis [all but 149 out of 54,832 from Schmid et al. (2005), submitted by TAIR] or maize [all from Sekhon et al. (2011), submitted by MaizeGDB]. Both studies used replicated microarray data that were collected as part of gene expression atlas studies. Arabidopsis data came from plants from the Columbia (Col-0) background, but some were wild type, while others are mutants (more details in Supplementary Table S1 in Schmid et al., 2005). Maize data came from plants from inbred line B73 (more details in Sekhon et al., 2011). We included only annotations with the evidence codes Inferred by Expression Pattern (IEP) and Inferred by Direct Assay (IDA) (Chibucos et al., 2014, 2017; Gene Ontology Consortium, 2015), which reflect the two kinds of experimental evidence that make up the bulk of PO-associated data. All data used in and generated by this analysis as well as a step-by-step description of methods used are available online^5^.

#### Assignment to Homolog Clusters

Plant Ontology annotations are linked to the gene or gene model^6^ names of a particular species. To allow for interspecific comparisons of similar sequences, we pooled all Arabidopsis and maize genes directly annotated to any *plant embryo development stage* in the PO and assigned them homolog cluster IDs based on the clusters determined by a customized adoption of InParanoid-based (Östlund et al., 2010) gene family clustering method (Shulaev et al., 2011; Myburg et al., 2014). InParanoid determines gene cluster homology using a reciprocal best match with BLAST. We used only homologs with a sequence overlap of 0.50, meaning that at least half of the two sequences must overlap each other, corresponding to a confidence cutoff of ∼0.05.

#### Calculating Unique Genes and Overlap Among Stages

Within each species, we calculated overlap of annotations between all possible pairs of stages using the gene IDs. We also calculated overlap using homolog cluster IDs, to examine the effect of pooling genes based on gene families. Between species, we compared all possible inter-species pairs of stages using only the lists of homolog cluster IDs. We calculated the approximate percent overlap in annotations between two stages (both within and between species) as

% overlap=(# annotations in common/average # of annotations)×100.

We also determined which genes were unique within each stage (did not occur in any other stage in the same species) and report both the number and percent of unique genes annotated to a single stage.

#### Analysis of GO Term Enrichment

To examine if the sets of genes associated to particular PO stages were related to function and development, we analyzed gene sets from selected pairs of stages using Singular Enrichment Analysis (SEA) and cross-comparison of SEA (SEACOMPARE) in agriGO v.2 (Tian et al., 2017). SEA computes GO term enrichment by comparing how often the members of a set of genes are associated to different GO terms relative to a reference set of genes. We used the pre-calculated set “Arabidopsis genome locus (TAIR10)” in agriGO as a reference. Under “Advanced options,” we limited terms for comparison to the Plant GO slim. All other parameters were set to default (using Fisher as the statistical method, Yekutieli as the multi-test adjustment methods, and 5 as the minimum number of mapping entries). Parameter details are available through the software.

**FIGURE 5:**
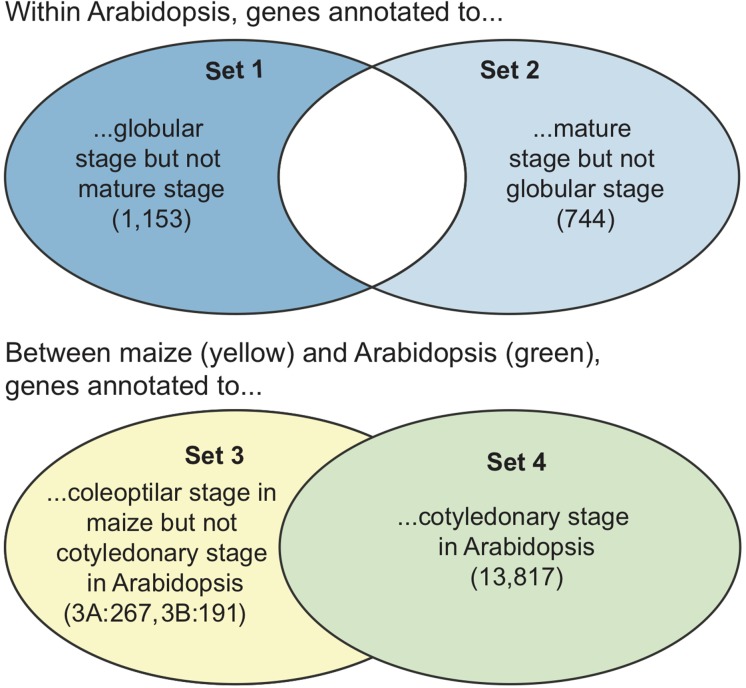
A graphical view of the sets of genes used in the GO term enrichment comparisons. Note that the final sets of genes only include those that are in the GO database, so the sets are slightly smaller than the input lists (see Supplementary Table S6). **(Top)** The sets of genes compared within Arabidopsis. Only non-overlapping sets were compared. **(Bottom)** The sets of genes (as maize gene IDs or Arabidopsis gene IDs converted from orthoIDs) compared between Arabidopsis and maize. The full set of Arabidopsis genes was compared to the non-overlapping genes from maize. Numbers in parentheses are the numbers of genes in each set. Set 3 was analyzed two ways, with maize gene IDs (set 3A) and with Arabidopsis gene IDs (set 3B).

Within Arabidopsis, we compared annotations for an early embryo development stage (*plant embryo globular stage*) to a late stage (*mature plant embryo stage*), to determine if the gross differences in anatomic development during these stages were associated with different patterns of gene function. We first generated a list of Arabidopsis genes annotated to *plant embryo globular stage* but not *mature plant embryo stage* (Set 1, Figure 5) as well as a set of genes annotated to *mature plant embryo stage* but not *plant embryo globular stage* (Set 2, Figure 5). We used SEA to get lists of enriched GO terms for Set 1 and Set 2, then used SEACOMPARE to visually compare the two lists of GO terms.

Across species, we compared the genes that were annotated to *plant embryo cotyledonary stage* (cotyledonary stage) in Arabidopsis to those that were annotated to *plant embryo coleoptilar stage* (coleoptilar stage) in maize. These stages, though unique to monocots or dicots, are both preceded by *plant embryo bilateral stage* (Figure 3). We used the list of homolog cluster IDs associated to the cotyledonary and coleoptilar stages to generate a list of homolog cluster IDs that were associated with the coleoptilar stage in maize, but not with the cotyledonary stage in Arabidopsis. In our original analysis, we converted the resulting set of homolog cluster IDs back to Arabidopsis gene identifiers to end up with a list of 191 Arabidopsis genes whose homologs have been associated with the coleoptilar stage of maize but which are not associated with the cotyledonary stage in Arabidopsis (Set 3B, Figure 5). While it seems more direct to compare GO enrichment of the Arabidopsis gene set to the maize gene set, this was not possible at the time, because maize gene annotations were not present in the GO. Later, maize gene annotations were added to GO, so we instead converted the homolog cluster IDs back to maize gene model identifiers to end up with a list of 267 maize gene models whose homologs have been associated with the coleoptilar stage of maize but which are not associated with the cotyledonary stage in Arabidopsis (Set 3A). In this paper, we present the results of both types of analyses, as shown on the right side of Figure 4. Direct analysis can be used whenever there are annotation data for a species in the GO database, and conversion to Arabidopsis gene IDs can be used for species that have no or limited annotation data in the GO database.

We used SEACOMPARE to compare the GO enrichment profiles of Set 3 genes to all genes annotated to the cotyledonary stage for Arabidopsis (Set 4, Figure 5). We did this two times – once to compare set 3A (with maize gene model IDs) to set 4, and once to compare set 3B (with Arabidopsis gene IDs) to set 4.

## Results

### Ontology Description and Major Revisions to Development Stage Terms

#### Accessing the Plant Ontology and Associated Annotation Data

The most recent version of the Plant Ontology is available through the Planteome browser^7^, as well as at the OBO Foundry website^8^, through BioPortal^9^, the Ontology Lookup Service^10^ and various other ontology listing services. The latest stable ontology files can also be accessed directly via permanent URLs^11^, and can be downloaded directly from the PO GitHub^12^ repository. Raw annotation files can be downloaded from the Planteome SVN repository^13^ or users can filter and download annotation data using the Planteome AmiGO browser annotation page^14^. A full description of the faceted searching features of the Planteome browser can be found in Cooper et al. (2018). Requests for additions or changes to the PO can be made by filing an issue on the PO GitHub issue tracker^15^. The latest statistics on the Plant Ontology (e.g., number of terms and properties) are available on the Planteome website^16^.

As of December 2018, PO users can query 1,086,153 associations between PO development stages and 100,728 unique data objects such as genes, gene models, and mutant phenotypes, across 15 species (Table 1). The largest contributors of annotations to the *plant structure development stage* terms are MaizeGDB (maize) and TAIR (Arabidopsis), with a large number for rice (*O. sativa*) developed by the PO curators and Gramene (Tello-Ruiz et al., 2016).

**Table 1:** Annotations to *plant structure development stage* terms by species and source in release version Nov 2017.

Species	Direct Annotations	# Bioentities	Source
*Zea mays* (maize)	818,816	38, 802	MaizeGDB^a^
*Arabidopsis thaliana*	186,750	18,947	TAIR^b^
*Oryza sativa* (rice)	61,226	32,395	Gramene^c^, POC^d^
*Solanum lycopersicum* (tomato)	11,074	4,318	SGN^e^
*Fragaria vesca* (strawberry)	5,657	3904	POC
*Vitis vinifera* (grape)	1,640	1420	POC, CRIBI^f^
*Solanum melongena* (eggplant)	540	235	SGN
*Glycine max* (soybean)	249	249	SoyBase^g^
*Gossypium hirsutum (cotton)*	161	123	SGN
*Capsicum annuum* (pepper)	15	15	SGN
*Petunia hybrida (Petunia)*	12	7	SGN
*Nicotiana tabacum* (tobacco)	7	4	SGN
*Coffea arabica (coffee)*	2	2	SGN
*Solanum pennellii*	2	1	SGN
*Solanum chmielewskii*	2	1	SGN
Total annotations:	1,086,153	100,728	

*^a^Maize Genetics and Genomics Database (http://www.maizegdb.org/). ^b^The Arabidopsis Information Resource (https://www.arabidopsis.org/). ^c^Gramene (http://www.gramene.org/). ^d^POC: Plant Ontology Consortium Curators (http://wiki.plantontology.org/index.php/Plant_Ontology_curators). ^e^Sol Genomics Network (http://solgenomics.net/) ^f^CRIBI: Grape Genome database: (http://genomes.cribi.unipd.it/grape/) ^g^SoyBase (http://soybase.org/).*

#### Organization and Scope of the *Plant Structure Development Stage* Branch of the PO

**FIGURE 6:**
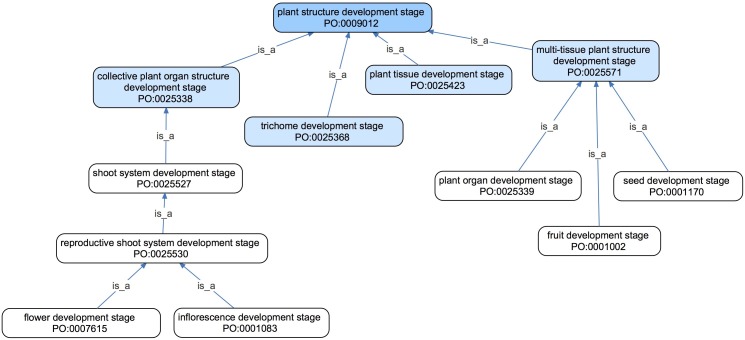
An ontology graph of selected terms in the *plant structure development stage* of the PO shown in their tree-like hierarchy using *is_a* or *subClassOf* relations (blue arrows). All direct subclasses of *plant structure development stage* are shown in light blue, with example subclasses of those terms shown in white. This hierarchy is similar to the hierarchy formed by the upper level classes of the *plant anatomical entity branch* in the PO (not shown).

*Plant structure development stage* is defined as a stage in the life of a *plant structure* during which the plant structure undergoes a *developmental process*, a term taken from GO. This branch of the PO encompasses stages in the development of any plant structure defined by the PO (Table 2), including the whole plant (Figure 1) and parts of plants, such as *flower development stage* (Table 2). *Plant structure development stage* classes are arranged in a hierarchy similar to that used for *plant structures* (Cooper et al., 2013). For example, a *flower development stage* is a *reproductive shoot system development stage*, which is a *shoot system development stage* (Figure 6).

**Table 2:** *Plant structure development stages* for specific plant parts that are not a *whole plant*, with sub-class hierarchy indicated by indents and >.

PO development stage	Identifier
**Multi-tissue plant structure development stage**	**PO:0025571**
>*fruit development stage*	PO:0001002
>*seed development stage*	PO:0001170
>*plant organ development stage*	PO:0025339
>>*root development stage*	PO:0007520
>>*phyllome development stage*	PO:0025579
>>>*leaf development stage*	PO:0001050
>>>*lemma development stage*	PO:0001047
>>>*lodicule development stage*	PO:0001049
>>>*palea development stage*	PO:0001048
**Collective plant organ structure development stage**	**PO:0025338**
>*shoot system development stage*	PO:0025527
>>*bud development stage*	PO:0025528
>>*reproductive shoot system development stage*	PO:0025530
>>>*flower development stage*	PO:0007615
>>>*inflorescence development stage*	PO:0001083
>*collective phyllome structure development stage*	PO:0025578
>> *anther development stage*	PO:0001004
>>*calyx development stage*	PO:0007603
>>*corolla development stage*	PO:0007604
>> *gynoecium development stage*	PO:0007606
>>>*ovule development stage*	PO:0007619
>>*androecium development stage*	PO:0007605
>>>*pollen development stage*	PO:0001007
**Plant tissue development stage**	**PO:0025423**
>*vascular tissue development stage*	PO:0025424
>>*secondary xylem development stage*	PO:0025427
>>*phloem development stage*	PO:0025426
>**Trichome development stage**	**PO:0025368**
>>*leaf trichome development stage*	PO:0007039
>>*seed trichome development stage*	PO:0025369

The PO currently does not represent stages for the development of *plant anatomical spaces*, because in most cases the development of spaces can be described by the development of surrounding structures. For example, the development of a *stomatal pore* is completely dependent upon the development of the *stomatal complex*. The PO also does not include development stages for subclasses of *portion of plant substance* such as *plant cuticle*, because plant substances do not undergo developmental processes, but rather are produced by *plant anatomical structures*.

#### Development Stages of Whole Plants

A *whole plant development stage* is a development stage of an entire plant, rather than one of its parts. Taken together, these stages make up the plant life cycle (Figure 2). *Whole plant development stage* has three direct subclasses: *sporophyte development stage*, *gametophyte development stage*, and *life of whole plant* (Figure 1). Both the *sporophyte* and *gametophyte development stages* have subclasses for vegetative, reproductive, dormant, and senescent stages (Figure 1, 2). These classes are subdivided further, e.g., *sporophyte vegetative stage* has subclasses such as *plant embryo development stage* and *seedling development stage*, each of which also has more specific subclasses. In addition, *sporophyte development stage* has a subclass for *plant zygote stage*, and *gametophyte development stage* has a subclass for *plant spore stage*. Zygote and spore stages are separate from the corresponding vegetative stages, because they each consist of a single cell whose existence precedes vegetative growth.

The PO term *life of whole plant* encompasses the whole life of any individual plant. Every instance of *life of whole plant* begins with meiosis, fertilization, or clonal reproduction (e.g., *in vitro* cultivation from a single cell) and ends with death. Every *gametophyte development stage* and *sporophyte development stage* is thus *part of* some *life of whole plant* (Figure 1) and *life of whole plant* currently has no subclasses. The *life of whole plant* term can be used in annotations to describe, e.g., a mutant with a shortened lifespan. It would, however, be more precise to describe which specific development stage is shorter, if known.

#### Development Stages of Individual Plant Parts

The PO includes development stage terms for plant parts such as flowers or leaves, plus a number of upper-level terms, such as *collective plant organ structure development stage*, used as grouping classes (Table 2). Newer classes include *secondary xylem development stage* and *seed trichome development stage* and their subclasses, which were added to facilitate descriptions of the development of woody species such as *Eucalyptus grandis* (Myburg et al., 2014) and *V. vinifera* (Fasoli et al., 2012), or plants with seed trichomes, such as cotton fibers in *Gossypium* spp. (Li et al., 2014; Dou et al., 2014).

#### Mapping PO Development Stage Terminology to Other Vocabularies

To facilitate annotation of data with PO terms, PO development stage terminology is mapped to existing vocabularies for Arabidopsis (Boyes et al., 2001), maize (Weber and Bleiholder, 1990; Lancashire et al., 1991), rice (*O. sativa*, Lancashire et al., 1991), cereals (wheat *Triticum* and *Aegilops* sp. L., barley *Hordeum vulgare* L., oat *Avena sativa* L., and rye *Secale cereale* L., Witzen-Berger et al., 1989; Lancashire et al., 1991), and grape (*V. vinifera*, Lorenz et al., 1995) – the latter four coming from the BBCH. Species- or clade-specific vocabularies typically use terms that are more granular than those in PO, so it is often the case that more than one stage from such a vocabulary will map to a single PO term. Because the major purpose of the PO is cross-species comparisons, we have not included many of the more granular terms that are specific to certain species, but prefer to maintain instead the mappings and allow taxon-specific vocabularies to provide the more granular terms in most cases.

### Comparison of Gene Expression During Embryo Development Reveals Few Stage-Specific Genes

#### Distribution of Annotations Among Development Stages

In the Planteome database, there were 15,078 unique Arabidopsis genes and 29,782 unique maize genes annotated via gene temporal expression to the subclasses of *plant embryo development stage*. Arabidopsis had annotations for *plant embryo globular stage, plant embryo bilateral stage*, *plant embryo cotyledonary stage* (which does not occur in grasses) and *mature plant embryo stage*. Maize had annotations for *plant proembryo stage*, *plant embryo coleoptilar stage* (which only occurs in grasses), and *plant embryo true leaf formation stage* (Figure 3). There were only 10 genes annotated to *plant proembryo stage* for Arabidopsis, so we excluded this species/stage combination from analysis. Differences in associations between species were due not only to differences in monocot and dicot development, but also to idiosyncrasies of the experiments that were used to generate the annotation data. There were between 13,739 and 28,663 annotations to each species/stage combination that we used (Table 3). A total of 10,397 unique Arabidopsis genes and 7,194 unique maize genes matched 8,182 unique gene homolog clusters with identities >0.50 (Table 3 and Supplementary Table S1).

**Table 3:** Plant Ontology annotations to plant embryo development stages for Arabidopsis and maize.

PO term	PO identifier	Annotated genes^a^	Homolog clusters^b^
**Arabidopsis:**			
*Plant embryo globular stage*	PO:0001185	13739	7805
*Plant embryo bilateral stage*	PO:0004507	13798	7855
*Plant embryo cotyledonary stage*	PO:0001078	13898	7906
*Mature plant embryo stage*	PO:0001081	13319	7691
**Maize:**			
*Plant proembryo stage*	PO:0001180	27117	7011
*Plant embryo coleoptilar stage*	PO:0001094	25065	6878
*Plant embryo true leaf formation stage*	PO:0001095	28663	7063

*^a^“Annotated genes” refers to the number of genes or gene models annotated directly to each stage. ^b^“Homolog clusters” refers to the number of annotations on a stage that match an Arabidopsis ortholog family (see section “Materials and Methods”).*

#### Genes Unique to Single Development Stages and Comparisons Among Stages

Within species, there were relatively few genes that were unique to a single *plant embryo development stage*, ranging from <1% to 7.57% (diagonal, Tables 4, 5). In Arabidopsis, the approximate percent overlap between pairs of stages varied from 92.53 to 97.31% for gene annotations and from 96.48 to 98.67% for homolog clusters (Table 4). In maize, the approximate percent overlap between pairs of stages varied from 92.32 to 94.17% for gene annotations and from 85.85 to 97.83% for homolog clusters (Table 5).

**Table 4:** Approximate percent overlap of expressed genes among Arabidopsis embryonic stages (light gray, above diagonal), approximate percent overlap of expressed homolog clusters (dark gray, below diagonal), and percent unique genes and (total gene counts) in each stage (blue, diagonal); see Section “Materials and Methods” for calculations of values.

PO Term	*Plant embryo globular stage* (PO:0001185)	*Plant embryo bilateral stage* (PO:0004507)	*Plant embryo cotyledonary stage* (PO:0001078)	*Mature plant embryo stage* (PO:0001081)	
*Plant embryo globular stage*	15.68	92.97	92.53	92.95	
(PO:0001185)	(781)				
*Plant embryo bilateral stage*	96.62	0.97	97.31	96.97	**Genes**
(PO:0004507)		(134)			
*Plant embryo cotyledonary stage*	96.37	98.67	1.77	96.61	
(PO:0001078)			(246)		
*Mature plant embryo stage*	96.48	98.43	98.24	0.36	
(PO:0001081)				(48)	
	**Homolog clusters**				

**Table 5:** Approximate percent overlap of expressed genes among maize embryonic stages (light gray, above diagonal), approximate percent overlap of expressed homolog clusters (dark gray, below diagonal), and percent unique genes and (total gene counts) in each stage (blue, diagonal); see Section “Materials and Methods” for calculations of values.

PO Term	*Plant proembryo stage* (PO:0001180)	*Plant embryo coleoptilar stage* (PO:0001094)	*Plant embryo true leaf formation stage* (PO:0001095)	
*Plant proembryo stage*	3.15	94.17	93.38	
(PO:0001180)	(855)			**Genes**
*Plant embryo coleoptilar stage*	85.85	0.19	92.32	
(PO:0001094)		(47)		
*Plant embryo true leaf formation stage*	97.83	86.18	7.57	
(PO:0001095)			(2169)	
	**Homolog clusters**			

Between species (Table 6), there was less overlap between pairs of stages than within species, ranging from 88.87 to 92.06%. Pooling across all stages within each species, 8,182 homolog clusters were present in Arabidopsis and 7,194 in maize, meaning that 988 homolog clusters were present in Arabidopsis but not maize.

**Table 6:** Approximate percent overlap of expressed genes in Plant Ontology annotations converted to homolog clusters (see section “Materials and Methods”), between selected pairs of *plant embryo development stages* in Arabidopsis and maize.

	Maize		
Arabidopsis	*Plant proembryo stage* (PO:0001180)	*Plant embryo coleoptilar stage* (PO:0001094)	*Plant embryo true leaf formation stage* (PO:0001095)
*Plant embryo globular stage*	91	89.61	88.87
(PO:0001185)			
*Plant embryo bilateral stage*	91.58	89.68	91.88
(PO:0004507)			
*Plant embryo cotyledonary stage*	91.75	89.84	92.06
(PO:0001078)			
*Mature plant embryo stage*	91.04	88.87	93.57
(PO:0001081)			

#### Comparison of GO Term Enrichment of Genes Unique to Early and Late Embryo Development Stages

Within Arabidopsis, there were 1,153 genes annotated to *plant embryo globular stage* but not *mature plant embryo stage* present in the GO database (Set 1, Figure 5, top). Compared to the Arabidopsis gene locus background, these genes were significantly over-represented in 41 GO terms (Supplementary Table S2 – Set 1). There were 744 Arabidopsis genes annotated to *mature plant embryo stage* but not *plant embryo globular stage* present in the GO database (Set 2, Figure 5, top), and these genes were significantly over-represented in 37 GO terms (Supplementary Table S2 – Set 2). Across both sets, there were 65 significantly enriched GO terms, 43 of which were biological processes, 13 molecular functions, and 8 cell components. There were only 13 GO terms that were over-represented in both Sets 1 and 2, including GO biological processes *developmental process*, *response to stimulus*, and *transcription factor activity, sequence-specific DNA binding*. Among the GO terms that were over-represented in the genes unique to the globular stage (Set 1), the most significant were GO biological processes *multi-organism process*, *response to stimulus*, and *carbohydrate metabolic process*, and GO cell components *extracellular region*, *external encapsulating structure*, *cell wall*, and *plasma membrane*. Among the GO terms that were over-represented in the genes unique to the mature stage (Set 2), the most significant were GO molecular function *DNA binding*, GO biological processes *regulation of cellular process* and *regulation of biological process*, and GO cellular component *nucleus*.

#### GO Term Enrichment of Homologs Annotated to Cotyledonary Stage but Not Coleoptilar Stage

There were 191 maize genes associated with the *plant embryo coleoptilar stage* in the PO but not associated with the *plant embryo cotyledonary stage* and 267 Arabidopsis genes whose orthologs have been associated with the *plant embryo coleoptilar stage* of maize in the PO but which have not been associated with the *plant embryo cotyledonary stage* in Arabidopsis (Set 3, Figure 5, bottom). In comparison, there were 13,817 genes associated with the *plant embryo cotyledonary stage* in Arabidopsis in the PO (Set 4, Figure 5, bottom).

Against the Gramene release 50 locus ID v3.30 background, set 3A genes (those with maize gene model IDs) were significantly over-represented in only two GO terms: GO biological processes *secondary metabolic process* and *carbohydrate metabolic process*. Of these two, only *carbohydrate metabolic process* was also significantly over represented for the genes associated with *plant embryo cotyledonary stage* (Set 4; Supplementary Table S3).

Against the Arabidopsis gene locus background, Set 3B genes (with Arabidopsis gene IDs) were significantly over-represented in 15 GO terms, all but two, *pollination* and *extracellular region*, were also over-represented for the genes associated with *plant embryo cotyledonary stage* (Set 4; Supplementary Table S4).

## Discussion

Plant Ontology terms for *plant structure development stages* have undergone extensive development and re-organization to broaden their application across land plants, with consideration for future expansion to include all green plants (land plants, plus the algae Chlorophyta and Charophyta). These changes make the PO more suitable for cross-taxon data integration and analysis. As genomic and phenomic data become available for more species and sampled with more precision, PO terminology will continue to evolve and provide the framework for efficient automated comparisons across species.

Note that several other ontologies or vocabularies exist for describing plants, and they can be used in conjunction with the Plant Ontology. The Plant Trait Ontology (Cooper et al., 2018), part of the Planteome suite of reference ontologies, describes plant traits and phenotypes. The Crop Ontology (CO^17^; Shrestha et al., 2011) is not actually an individual ontology, but a collection of approximately 40 species-specific vocabularies with very limited semantics, covering germplasm, phenotype or trait, and location and environment. The Common Agricultural Vocabulary (CAVOC; Joo et al., 2018) is a Japanese vocabulary system of agricultural crop names developed for the purpose of data cooperation between agricultural systems, presented in Japanese. The Agronomy Ontology (Devare et al., 2016) describes agronomic practices, techniques, and variables used in agronomic experiments.

### Creating Development Stage Terms That Apply Across All Land Plants

A major challenge in creating a development stage ontology for all plant species is to define stages that are broad enough to cover the life cycle of all plants, yet detailed enough to meet the needs of annotators working on particular species. Translating the life cycle of semelparous, determinate, annual angiosperms (i.e., the classic model plant species) into well-defined stages that worked for multiple species was the task of the original GSO (Pujar et al., 2006). The task of defining *whole plant development stages* that also work across perennial, iteroparous, and indeterminate plants presented new challenges.

We chose to define the vegetative, reproductive, and senescent stages as occurring sequentially. Under this representation, a *sporophyte reproductive stage* occurs during the interval between the initiation of a sporangium and the onset of senescence. Once a plant produces a single sporangium, it remains in the *sporophyte reproductive stage* until it begins to senesce and does not cycle back and forth between a *sporophyte reproductive stage* and a *sporophyte vegetative stage*, even though an iteroparous plant may well cycle back and forth between reproductive and vegetative growth. A similar simplification is used for the *gametophyte vegetative* and *reproductive stages* (Figure 2). Although this classification may seem unintuitive for iteroparous plants, it is important to remember that the PO’s high-level classes for vegetative and reproductive stages are intended to describe plant development, which is a linear process, not phenology, which is cyclic. We do not expect that annotations for gene expression will be associated with these high level classes, which serve primarily as grouping categories for more specific classes. This allows users to search, for example, for all annotations on both *whole plant flowering stage* and *whole plant fruit development stage* by searching on *sporophyte reproductive stage*. Researchers wanting to annotate data to flowering or fruiting stages can use the more specific PO classes, which are logically consistent with both semelparous and iteroparous plant development as well as the linear development of an entire plant (Figure 2). Researchers needing to describe the development of part of a plant, such as an individual branch, a flower, or a trichome, can use PO stage terms for the specific structure (Table 2). For researchers needing to more precisely describe the phenological stage of a plant or plant part, the Plant Phenology Ontology (PPO; Stucky et al., 2018) is now available. PPO uses PO terms to define its classes for phenological traits such as *flowers present* or *senescing true leaves absent*.

The hierarchical nature of PO allows it to retain some specific stages for annual crop and model species that were developed as part of the original GSO, but nest them within more general stages for all species. For example, *whole plant fruit formation stage* has subclasses such as *whole plant fruit formation stage 30–50%* that are best used for plants with more or less synchronous fruit development where the final size of the fruit is known, such as some maize cultivars. They are not appropriate for species that have indeterminate growth and asynchronous fruit ripening, such as some tomato cultivars. Nonetheless, indeterminate tomato development stages can be annotated using PO terminology by associating whole plant development to more general terms like *whole plant fruit formation* or *whole plant fruit ripening stage*, with more specific associations to terms for development stages of individual fruits, such as the subclasses of *fruit development stage*.

#### Dormancy and Senescence

Plant Ontology terms for *gametophyte-* and *sporophyte dormant stage* are defined as occurring whenever a plant is participating in a GO:*dormancy process*, which may occur more than once during a *life of whole plant*. During a dormant stage, some organs may senesce, but parts of the plant remain alive (e.g., the woody *shoot system* or the *root system*). We chose this model, because plant dormancy is not restricted to a particular part of the plant life cycle and often occurs at different stages of development in different species. Thus, in contrast to the treatment of vegetative and reproductive stages, the PO does allow an individual plant to enter into and out of a *gametophyte* or *sporophyte dormant stage* multiple times during its life.

Likewise, a plant may begin a *gametophyte* or *sporophyte senescent stage* from either a vegetative or reproductive stage, but in contrast to dormancy, an individual plant can have only one *gametophyte* or *sporophyte senescent stage*. Although organs within the plant may senesce at various points during the plant’s life, once a whole plant enters a *gametophyte* or *sporophyte senescent stage*, it can only exit it by dying.

#### Developmental Processes Versus Developmental Stages

For interoperability with other ontologies in the OBO Foundry (Smith et al., 2007), the PO is rooted in the Basic Formal Ontology (BFO, Grenon and Smith, 2004; Arp et al., 2015). *Plant structure development stage* is a subclass of BFO:*occurrent*, although this relation is not explicit in the PO ontology file. *Plant structure development stages* in the PO are distinct from the sorts of *occurrents* defined under *biological process* in the GO (Ashburner et al., 2000; The Gene Ontology Consortium, 2012), in that PO development stages are intervals of a plant’s life during which GO:*biological processes* occur. Processes – such as *reproduction* or *dormancy* – are incorporated into the PO by using the corresponding GO terms in PO definitions, as for instance in the case of *sporophyte reproductive stage* and *gametophyte dormant stage*. PO curators work with the GO curators to create or refine the developmental process terms needed to define PO plant development stages and ensure that GO definitions are appropriate for all land plants.

#### Development Stages for a Wider Range of Plant Structures

The growing pool of genotypic and phenotypic data for new model and non-model species motivates the creation of new PO terms to represent development stages of the many types of structures now being studied. These new terms also make it easier to extend the PO to new plant species. For example, classes for *seed trichome development stages* were added at the request of scientists describing gene expression in cotton, but because these stages have been described generically, they can be used for any plant with *seed trichomes*. PO curators continue to work with experts from different communities to incorporate new development stage terms, such as those for *secondary xylem* (wood) *development stages* (Lens et al., 2012). Work is ongoing to add new terms for flagellate plants, through a collaboration with the biodiversity community (Endara et al., 2018).

### Case Study: Comparison of Gene Expression Across Species and Stages

The comparison of developmental stages across species is complicated by different developmental patterns, such as those observed in monocotyledonous versus dicotyledonous species, which share the earliest stages of development before diverging into different developmental paths. Both monocot and dicot embryos go through a *plant proembryo stage*, a *plant embryo globular stage*, and a *plant embryo bilateral stage*. In dicots such as Arabidopsis, the developing embryo next goes through a *plant embryo cotyledonary stage* (synonym “torpedo stage”), whereas a monocot maize embryo passes into a *plant embryo coleoptilar stage.* Although not known to be homologous, *plant embryo cotyledonary stage* is similar to *plant embryo coleoptilar stage* in that both represent the early development of the first leaf-like structures. Therefore, the goal of this analysis was to determine if PO and GO annotations could reveal meaningful differences in expression patterns between these two stages that may point to the genetic basis of *cotyledon* versus *coleoptile* development.

Using the Planteome database, we identified a set of genes that are associated with the coleoptilar stage of maize but not the cotyledonary stage in Arabidopsis (Sets 3A and 3B). This list represents genes that may play a role in early grass development by contributing to the formation of a *coleoptile* as opposed to a *cotyledon*. Our intention was to demonstrate the query capabilities of the PO, rather than to robustly test developmental hypotheses, thus we make the list of genes available for further investigation to determine what role those genes may play in coleoptile development (Supplementary Table S5 lists all genes unique to one stage, Supplementary Table S6 lists the genes used to generate sets 1–4). GO enrichment analysis (Supplementary Tables S3, S4) indicated that these genes play a role in biological processes such as *transport* and *response to stimulus*, and nearly all of the GO terms enriched for Set 3A or 3B were also enriched for the set of genes expressed during the cotyledonary stage (Set 4). Given that Set 3 only shows significant enrichment in high-level terms, regardless of whether maize or Arabidopsis gene IDs are use, the result does not inform on if, or how, these genes may contribute to coleoptile development. Rather, the set may simply contain genes that have not yet been examined during the cotyledonary stage in Arabidopsis. While this particular result is inconclusive, our method represents a new way to assess gene expression and development in plant species outside Arabidopsis, for which GO annotations data are lacking. It is important to note the major differences between the analyses using maize versus Arabidopsis gene IDs (and the corresponding background annotation dataset in the GO), as these differences reveal how much the available data in the GO and PO databases can impact the results of an enrichment analysis.

Within a single species, approximate overlap among *plant embryo developmental stages* (Tables 4–6) was high and very similar to the average 92% found in distinct tissues by Schmid et al. (2005), suggesting that temporal variation in gene expression may be similar to spatial/tissue variation. However, high overlap does not necessarily indicate similar expression patterns among stages, because PO annotations do not convey the level or direction of expression. Unique expression patterns during different stages of Arabidopsis embryogenesis have in fact been demonstrated using cluster analysis (Xiang et al., 2011). High overlap does suggest that many of the genes involved in embryo development are either active throughout all embryo development stages or are general-purpose (e.g., house keeping) genes that are not specific to one development stage. This is further supported by our comparison of early and late embryo development stages in Arabidopsis, where terms such as *transcription factor activity, sequence specific DNA binding* or *response to stimulus* were over represented in both early and late stage gene sets (Supplementary Table S2). The notably different enrichment patterns between early and late embryo development stages observed by Xiang et al. (2011) do indicate functional variation in the sets of genes expressed over the course of development, meriting further study.

### Applications of PO *Plant Structure Development Stage* Terminology to Data Integration

Walls et al. (2012a) described the general utility of ontologies for plant sciences. In what follows, we describe advances that may arise specifically through the enhancements of PO *plant structure development stage* terms.

#### Plant Diseases

Many pests and pathogens attack plants during specific developmental stages, and infection or the appearance of symptoms may occur at different stages in different species. Farmers manage crop diseases by careful timing of planting, pesticide application, or harvest (Smith et al., 2004; Wise et al., 2012), and the original BBCH growth stages were created in part to enable consistency in agronomic practices. However, the BBCH specifies relationships among the stages for different species only in general categories that are too broad to be useful for most cross-species comparisons. The association of PO *plant structure development stages* to plant pathology data will help make it possible to explore and learn the molecular and environmental basis of plant diseases using advanced semantic methods (Walls et al., 2012b).

#### Phenotypes and Imaging

New methods for rapid plant phenotyping portend a flood of phenotypic data (Arvidsson et al., 2011; Furbank and Tester, 2011; Crain et al., 2016). Extracting knowledge from this new source of big data requires the application of computational methods, thus requiring that data are in a computable form. Best practices have been documented that enable generation of computable data through ontology-based annotation of plant genotypes and phenotypes (Oellrich et al., 2015; Ćwiek-Kupczyńska et al., 2016; ten Hoopen et al., 2016), and these standards include recording plant development stages. An analogous approach can be applied to images. Segmentation and labeling tools – such as AISO:Annotation of Images Segments with Ontologies (Lingutla et al., 2014) and Bisque (Kvilekval et al., 2010) – can be applied to image corpora to provide training sets for machine learning algorithms that can automatically classify images for different plant structures, development stages, and associated disease phenomena.

#### Climate Change and Biodiversity

Plant responses to climate change are often recorded as phenological changes in the timing of developmental stages, ultimately leading to demographic changes (Fitter and Fitter, 2002; Parmesan, 2006). However, the effects of climate change on phenology and demography can be difficult to document given the need for long-term data (Miller-Rushing et al., 2010). The growth of online resources (e.g., Rosemartin et al., 2014; Elmendorf et al., 2016) or the use of herbarium specimen data (Miller-Rushing et al., 2006) can help to overcome this challenge, but their utility is limited by the divergent terminologies used for describing the phenology. Ontologies such as the PO can play a central role in facilitating computational approaches to the study of large-scale phenological changes by providing not only a consistent, controlled vocabulary of development stages already in use across a broad range of data sources, but also the semantic structure to appropriately aggregate phenological data scored at different levels of precision. The recently published Plant Phenology Ontology (Stucky et al., 2018) uses the PO as a basis of its classification of plant phenological traits.

#### Comparative Development and Evolution

Genetic and genomic data for an ever-increasing set of green plant species can accelerate discoveries in developmental plant biology. These data are elucidating the genetic basis for key innovations in land plants (Bowman et al., 2007; Rensing et al., 2008), the degree to which genes or gene expression profiles are conserved across species, or how different developmental pathways leading to similar forms (e.g., Jiao et al., 2005; Johnson et al., 2006; Chandler et al., 2008). The incompatibility of data from different species remains a barrier to harvesting the full value from published data. Use of the PO can overcome this barrier by mapping development stage terms from different species to a common, shared vocabulary. For example, PO users interested in early inflorescence development can search for annotations to the PO term *whole plant inflorescence detectable stage* and its subclasses to find data from Arabidopsis, maize, and tomato, including data originally formulated using a wide range of synonymous terms for stages in these species. Existing PO data from model species could be used to predict the orthologous gene expression or mutant phenotypes of non-model species, as described in Oellrich et al. (2015).

### Conclusion and Future Developments

Comparison of developmental stages across species is inhibited by multiple factors, including different developmental patterns observed in different species (for example in monocots versus dicots or angiosperms versus bryophytes) and species-specific differences in community practices for collecting and formulating data. The PO makes it possible to highlight commonalities among plant species in a machine-readable way, thereby facilitating new sorts of comparisons and advancing interoperability of data, to allow aggregation on a scale hitherto impossible. With the addition of new *whole plant development stages* and modification of many of the subclasses of *sporophyte development stage*, the PO now can be used for annotating the entire life cycle of any land plant. The PO continues to expand and improve, with the ongoing addition of new components, refinement of existing components, and addition of new annotation data. The case study presented in this paper demonstrates how PO annotations can be used to identify candidate genes that may play a role in the development of different plant structures, such as the formation of a *coleoptile* as opposed to a *cotyledon*.

Future work on PO development stages should expand logical definitions, add taxon constraints that formalized restrictions of terms to specific taxa to improve annotation quality (Deegan (nee Clark) et al., 2010), and create more explicit links between PO development stages and GO developmental processes to enhance the reasoning power of both ontologies. Specific branches of the PO could be improved by further development and enrichment. For example, *fruit development stage*, which describes the development of individual fruits has only two subclass stages: *fruit formation stage* and *fruit ripening stage*. These broad stages could be specified more precisely for the different types of fruits in the PO. The plant sciences community still has plenty of work to do to develop ontologies and association data before their full potential can be realized, and new automated data mining methods can accelerate this process (e.g., Xu et al., 2016). There are many applications that could be enhanced today through the use of the Plant Ontology terminology for *plant structure development stages*, and the PO development team welcomes input for new and improved ontology terms and annotation data.

## Author Contributions

PJ and DS conceived the project. RW, LC, MG, CM, BS, DS, and PJ designed and created the PO. LC coordinated the project. JE, CM, and PJ designed and created the PO annotation database. RW led the design and comparative analysis of the embryo stage annotations. JE did the gene homology analysis. RW, LC, JE, and PJ interpreted the results of the analysis. RW and LC wrote the manuscript, with all authors contributing edits and review.

## Conflict of Interest Statement

The authors declare that the research was conducted in the absence of any commercial or financial relationships that could be construed as a potential conflict of interest. The reviewer RH declared a past co-authorship with one of the authors, CM, to the handling Editor.

## Footnotes

1 http://www.planteome.org

2 Throughout this paper, words in italics represent ontology terms (classes or relations), except where they represent the scientific name of a species.

3 http://wiki.plantontology.org/index.php/Relations_in_the_Plant_Ontology

4 http://planteome.org/svn/po-associations/?pathrev=412

5 https://github.com/Planteome/planteome-analyses/tree/master/embryo_stage_comparison

6 For brevity we henceforth use the term “gene” to refer to both known genes and gene models.

7 http://browser.planteome.org/amigo

8 http://www.obofoundry.org/

9 https://bioportal.bioontology.org/ontologies/PO

10 https://www.ebi.ac.uk/ols/ontologies/po

11 http://purl.obolibrary.org/obo/po.owl or http://purl.obolibrary.org/obo/po.obo

12 https://github.com/Planteome/plant-ontology

13 http://planteome.org/svn/po-associations

14 http://browser.planteome.org/amigo/search/annotation

15 https://github.com/Planteome/plant-ontology/issues

16 http://planteome.org/documents/release_notes/Planteome_Version_3.0_September_2018

17 http://www.cropontology.org/

## Supplementary Material

DOCUMENT S1 Creating PO annotations.

TABLE S1 Gene homolog clusters as generated by InParanoid-based analysis. Column headers are “id”, homolog cluster identifier generated by InParanoid; “gene”, standardized Arabidopsis gene identifier; “ortho_gene”, standardized maize gene model identifier of the ortholog that corresponds to the Arabidopsis gene; “score”, sequence overlap score from InParanoid.

TABLE S2 Comparison of GO enrichment analysis for Set 1 versus Set 2 genes in Arabidopsis. Set 1: Genes associated to *plant embryo globular stage* (*globular*) but not *mature plant embryo stage* (*mature*). Set 2: Genes associated to *mature plant embryo stage* (*mature*) but not *plant embryo globular stage* (*globular*). GO Branches: BP, *biological process*; MF, *molecular function*; and CC, *cellular component*. FDR is the False Discovery Rate of (Benjamini and Yekutieli, 2001). Any reported value is significant at *P* = 0.05 when corrected for multiple tests. Colors correspond to FDR values, with red most significant and dark gray non-significant.

TABLE S3 Comparison of GO across species for Set 3A and Set 4. Set 3A: Maize genes that have been associated with the *plant embryo coleoptilar stage* (coleoptilar) of maize in the PO, but which have not been associated with the *plant embryo cotyledonary stage* (cotyledonary) in Arabidopsis. Set 4: *G*enes associated with the *plant embryo cotyledonary stage* in Arabidopsis. GO Branches: BP, *biological process*; MF, *molecular function*; and CC, *cellular component*. FDR is the False Discovery Rate of (Benjamini and Yekutieli, 2001). Any reported value is significant at *P* = 0.05 when corrected for multiple tests. Colors correspond to FDR values, with red most significant and dark gray non-significant.

TABLE S4 Comparison of GO across species for Set 3B and Set 4. Set 3B: Arabidopsis genes whose orthologs have been associated with the *plant embryo coleoptilar stage* (coleoptilar) of maize in the PO, but which have not been associated with the *plant embryo cotyledonary stage* (cotyledonary) in Arabidopsis. Set 4: *G*enes associated with the *plant embryo cotyledonary stage* in Arabidopsis. GO Branches: BP, *biological process*; MF, *molecular function*; and CC, *cellular component*. FDR is the False Discovery Rate of (Benjamini and Yekutieli, 2001). Any reported value is significant at *P* = 0.05 when corrected for multiple tests. Colors correspond to FDR values, with red most significant and dark gray non-significant.

TABLE S5 List of genes or gene models annotated a single Plant Ontology plant embryo development stage.

TABLE S6 List of genes or gene models used as inputs to sets 1–4. These are the lists of input genes used in the analysis. The final sets of genes only include those that are in the GO database, so the sets are slightly smaller than the input lists.
