# Supplementary material for: The Plant Ontology Facilitates Comparisons of Plant Development Stages Across Species
Source: Front Plant Sci. 2019 Jun 4;10:631. doi: 10.3389/fpls.2019.00631 (PMC6558174; doi:10.3389/fpls.2019.00631)
Supplement: DOCUMENT S1 — Creating PO annotations. [file Data_Sheet_1.PDF]

Supplemental Document S1: Creating PO annotations.

Methods

Annotations can be created and submitted by anyone, but most annotations in the Plant Ontology (PO) database are created by curators and model organism databases (DB) such as TAIR and MaizeGDB, then submitted to the PO for ingestion. The process – described in the follow steps – is a collaboration between DB curators, the original study authors, and PO curators.

1. DB curator selects an appropriate study for annotation [eg., (Li *et al.*, 2010; Sekhon *et al.*, 2011; Fasoli *et al.*, 2012; Kang *et al.*, 2013)].
2. DB curator creates a mapping table between the terms used in the study and PO terms and a table of sources. Mappings are created in consultation with study authors and PO curators as needed. This is usually done using collaborative editing software such as Google Docs or Dropbox.
3. PO curators add any needed terms to the ontology and fix any issues with existing terms and definitions.
4. PO curators create an appropriate database cross-reference (dbxRef) to the DB database holding the expression data, if one does not exist. A dbxRef is a code used in the annotation database that is converted to hyperlink to the DB in the PO browser.
5. DB curator creates a 16-column spreadsheet following the gene associations file (GAF 2.0) format (http://www.geneontology.org/GO.format.annotation.shtml). This spreadsheet is listing of the PO identifiers, the associated genes, gene models, proteins etc., the dbxRef and other information. Annotations for plant anatomy terms (*plant anatomical structures*) are kept in a separate table from annotations for *plant structure development stages*.
6. A tab delimited text file version of the spreadsheet is upload to the PO SVN repository at http://palea.cgrb.oregonstate.edu/viewsvn/Poc/trunk/associations/.
7. PO curators integrate the new annotations into the PO database and check it against the most current version of the PO.
8. New annotations are made public when a new version of the PO is released.

Discussion

PO annotations the GO model and adhere to the GAF format as described in more detail in the PO wiki section on annotations. There are two major components to annotation: a link between a gene product and a descriptive PO definition and data about the source and evidence used to make the link. Each annotation must include a reference. This is usually a PubMed ID (PMID) linking back to the original scientific paper describing the study used for annotation. The type of evidence used to make an annotation is recorded in the evidence code column.

Literature cited:

Fasoli M, Dal Santo S, Zenoni S, Tornielli GB, Farina L, Zamboni A, Porceddu A, Bicego M, Murino V, Ferrarini A, *et al.* 2012. The grapevine expression atlas reveals a deep transcriptome shift driving the entire plant into a maturation program. *The Plant cell* **24**: 3489–3505.

Kang C, Darwish O, Geretz A, Shahan R, Alkharouf N, Liu Z. 2013. Genome-scale transcriptomic insights into early-stage fruit development in woodland strawberry Fragaria vesca. *The Plant Cell* **25**: 1960–1978.

Li P, Ponnala L, Gandotra N, Wang L, Si Y, Tausta SL, Kebrom TH, Provart N, Patel R, Myers CR, *et al.* 2010. The developmental dynamics of the maize leaf transcriptome. *Nature Genetics* **42**: 1060–1067.

Sekhon RS, Lin H, Childs KL, Hansey CN, Buell CR, de Leon N, Kaeppler SM. 2011. Genome-wide atlas of transcription during maize development. *The Plant Journal* **66**: 553–563.
