# Supplementary material for: The Plant Ontology Facilitates Comparisons of Plant Development Stages Across Species
Source: Front Plant Sci. 2019 Jun 4;10:631. doi: 10.3389/fpls.2019.00631 (PMC6558174; doi:10.3389/fpls.2019.00631)
Supplement: TABLE S2 — Comparison of GO enrichment analysis for Set 1 versus Set 2 genes in Arabidopsis. Set 1: Genes associated to plant embryo globular stage (globular) but not mature plant embryo stage (mature). Set 2: Genes associated to mature plant embryo stage (mature) but not plant embryo globular stage (globular). GO Branches: BP, biological process; MF, molecular function; and CC, cellular component. FDR is the False Discovery Rate of (Benjamini and Yekutieli, 2001). Any reported value is significant at P = 0.05 when corrected for multiple tests. Colors correspond to FDR values, with red most significant and dark gray non-significant. [file Table_2.pdf]

GO Information			FDR	
GO Term	Onto	Description	Set 1 - globular not mature	Set 2 - mature not globular
GO:0051704	P	multi-organism process	1.1E-09	---
GO:0050896	P	response to stimulus	0.000000013	0.01
GO:0005975	P	carbohydrate metabolic process	0.000000032	---
GO:0006950	P	response to stress	0.00001	---
GO:0009607	P	response to biotic stimulus	0.000029	---
GO:0009856	P	pollination	0.000046	---
GO:0009056	P	catabolic process	0.00013	---
GO:0003006	P	developmental process involved in reproduction	0.00024	0.033
GO:0019748	P	secondary metabolic process	0.00026	---
GO:0009719	P	response to endogenous stimulus	0.00048	0.017
GO:0009605	P	response to external stimulus	0.00055	---
GO:0022414	P	reproductive process	0.00063	0.0013
GO:0006810	P	transport	0.00063	---
GO:0000003	P	reproduction	0.00067	0.0013
GO:0048869	P	cellular developmental process	0.00069	---
GO:0051234	P	establishment of localization	0.00069	---
GO:0007154	P	cell communication	0.00094	0.000072
GO:0051179	P	localization	0.00094	---
GO:0007165	P	signal transduction	0.0011	0.000037
GO:0032502	P	developmental process	0.0014	0.0058
GO:0048856	P	anatomical structure development	0.0018	0.005
GO:0030154	P	cell differentiation	0.0022	0.034
GO:0032501	P	multicellular organismal process	0.003	0.002
GO:0008152	P	metabolic process	0.0086	---
GO:0065007	P	biological regulation	0.015	0.0000066
GO:0016049	P	cell growth	0.016	---
GO:0040007	P	growth	0.018	---
GO:0006629	P	lipid metabolic process	0.042	---
GO:0005215	F	transporter activity	0.00000082	---
GO:0003824	F	catalytic activity	0.000011	---
GO:0003700	F	transcription factor activity, sequence-specific DNA binding	0.00013	0.0000042
GO:0008289	F	lipid binding	0.0065	---
GO:0016787	F	hydrolase activity	0.0072	---
GO:0030234	F	enzyme regulator activity	0.045	---
GO:0005576	C	extracellular region	7.9E-21	---
GO:0030312	C	external encapsulating structure	3.6E-10	---
GO:0005618	C	cell wall	3.6E-10	---
GO:0005886	C	plasma membrane	1.6E-09	---
GO:0016020	C	membrane	0.036	---
GO:0044421	C	extracellular region part	0.036	---
GO:0031012	C	extracellular matrix	0.036	---
GO:0050794	P	regulation of cellular process	---	0.0000002
GO:0050789	P	regulation of biological process	---	0.00000034
GO:0060255	P	regulation of macromolecule metabolic process	---	0.000001
GO:0010468	P	regulation of gene expression	---	0.0000038
GO:0006139	P	nucleobase-containing compound metabolic process	---	0.0000038
GO:0019222	P	regulation of metabolic process	---	0.0000043
GO:0009908	P	flower development	---	0.0001
GO:0006259	P	DNA metabolic process	---	0.00019
GO:0007049	P	cell cycle	---	0.0016
GO:0007275	P	multicellular organism development	---	0.0071
GO:0009653	P	anatomical structure morphogenesis	---	0.0083
GO:0009791	P	post-embryonic development	---	0.0086
GO:0044260	P	cellular macromolecule metabolic process	---	0.0091
GO:0048608	P	reproductive structure development	---	0.014
GO:0043170	P	macromolecule metabolic process	---	0.033
GO:0009987	P	cellular process	---	0.033
GO:0003677	F	DNA binding	---	0.000000044
GO:0005488	F	binding	---	0.035
GO:0016301	F	kinase activity	---	0.035
GO:0016772	F	transferase activity, transferring phosphorus-containing groups	---	0.035
GO:0030246	F	carbohydrate binding	---	0.035
GO:0003676	F	nucleic acid binding	---	0.043
GO:0019825	F	oxygen binding	---	0.048
GO:0005634	C	nucleus	---	0.0000047
