# Supplementary material for: The Plant Ontology Facilitates Comparisons of Plant Development Stages Across Species
Source: Front Plant Sci. 2019 Jun 4;10:631. doi: 10.3389/fpls.2019.00631 (PMC6558174; doi:10.3389/fpls.2019.00631)
Supplement: TABLE S4 — Comparison of GO across species for Set 3B and Set 4. Set 3B: Arabidopsis genes whose orthologs have been associated with the plant embryo coleoptilar stage (coleoptilar) of maize in the PO, but which have not been associated with the plant embryo cotyledonary stage (cotyledonary) in Arabidopsis. Set 4: Genes associated with the plant embryo cotyledonary stage in Arabidopsis. GO Branches: BP, biological process; MF, molecular function; and CC, cellular component. FDR is the False Discovery Rate of (Benjamini and Yekutieli, 2001). Any reported value is significant at P = 0.05 when corrected for multiple tests. Colors correspond to FDR values, with red most significant and dark gray non-significant. [file Table_4.pdf]

GO Information			FDR	
GO Term	Onto	Description	Set 3B - coleoptilar not cotyledonary - arab. IDs	Set 4 - cotyledonary
GO:0005975	P	carbohydrate metabolic process	0.0016	0.0000076
GO:0009719	P	response to endogenous stimulus	0.002	6.6E-11
GO:0009056	P	catabolic process	0.002	0.000000014
GO:0006810	P	transport	0.01	1.6E-17
GO:0051234	P	establishment of localization	0.01	9.9E-18
GO:0051179	P	localization	0.011	2.6E-18
GO:0009856	P	pollination	0.05	---
GO:0003824	F	catalytic activity	0.000055	2E-55
GO:0005215	F	transporter activity	0.0011	0.0000004
GO:0016788	F	hydrolase activity, acting on ester bonds	0.012	0.0000018
GO:0005886	C	plasma membrane	0.013	2E-22
GO:0005576	C	extracellular region	0.013	---
GO:0030312	C	external encapsulating structure	0.032	0.007
GO:0005618	C	cell wall	0.032	0.007
GO:0016020	C	membrane	0.037	4.6E-44
GO:0009987	P	cellular process	---	3.1E-77
GO:0044237	P	cellular metabolic process	---	6.2E-50
GO:0008152	P	metabolic process	---	2E-46
GO:0044238	P	primary metabolic process	---	2.9E-37
GO:0016043	P	cellular component organization	---	1.3E-30
GO:0050896	P	response to stimulus	---	5.1E-28
GO:0009628	P	response to abiotic stimulus	---	9.7E-28
GO:0006139	P	nucleobase-containing compound metabolic process	---	1.2E-27
GO:0050789	P	regulation of biological process	---	8.4E-27
GO:0032502	P	developmental process	---	1E-26
GO:0048856	P	anatomical structure development	---	2.9E-25
GO:0050794	P	regulation of cellular process	---	3.5E-23
GO:0007275	P	multicellular organism development	---	4.5E-23
GO:0032501	P	multicellular organismal process	---	5.9E-23
GO:0009791	P	post-embryonic development	---	1.6E-21
GO:0043412	P	macromolecule modification	---	2.9E-19
GO:0044260	P	cellular macromolecule metabolic process	---	5E-19
GO:0043170	P	macromolecule metabolic process	---	5.5E-19
GO:0048608	P	reproductive structure development	---	1.2E-17
GO:0065007	P	biological regulation	---	4.3E-17
GO:0006464	P	cellular protein modification process	---	5.4E-17
GO:0000003	P	reproduction	---	1.7E-16
GO:0006807	P	nitrogen compound metabolic process	---	2E-16
GO:0022414	P	reproductive process	---	2.9E-16
GO:0003006	P	developmental process involved in reproduction	---	4.2E-15
GO:0019222	P	regulation of metabolic process	---	5.3E-12
GO:0006950	P	response to stress	---	2.6E-11
GO:0044249	P	cellular biosynthetic process	---	6.6E-11
GO:0009058	P	biosynthetic process	---	1.7E-10
GO:0006259	P	DNA metabolic process	---	2.1E-10
GO:0009790	P	embryo development	---	8.3E-10
GO:0006091	P	generation of precursor metabolites and energy	---	8.3E-10
GO:0015979	P	photosynthesis	---	8.5E-10
GO:0007049	P	cell cycle	---	8.5E-10
GO:0060255	P	regulation of macromolecule metabolic process	---	4.3E-09
GO:0009908	P	flower development	---	5.8E-09
GO:0009653	P	anatomical structure morphogenesis	---	0.000000007
GO:0007165	P	signal transduction	---	0.00000005
GO:0007154	P	cell communication	---	0.00000023
GO:0010468	P	regulation of gene expression	---	0.00000072
GO:0006629	P	lipid metabolic process	---	0.000001
GO:0019538	P	protein metabolic process	---	0.000088
GO:0040007	P	growth	---	0.000098
GO:0044267	P	cellular protein metabolic process	---	0.00027
GO:0048869	P	cellular developmental process	---	0.00031
GO:0042592	P	homeostatic process	---	0.00032

GO:0019725	P	cellular homeostasis	---	0.0013
GO:0030154	P	cell differentiation	---	0.019
GO:0008219	P	cell death	---	0.02
GO:0016049	P	cell growth	---	0.021
GO:0010467	P	gene expression	---	0.035
GO:0005515	F	protein binding	---	4.3E-57
GO:0005488	F	binding	---	7E-56
GO:0000166	F	nucleotide binding	---	3.8E-29
GO:0016787	F	hydrolase activity	---	1.3E-17
GO:0016740	F	transferase activity	---	3.5E-17
GO:0016817	F	hydrolase activity, acting on acid anhydrides	---	1.8E-11
GO:0016818	F	hydrolase activity, acting on acid anhydrides, in phosphorus-containing anhydrides	---	1.9E-11
GO:0016462	F	pyrophosphatase activity	---	2.4E-11
GO:0017111	F	nucleoside-triphosphatase activity	---	3.3E-10
GO:0016772	F	transferase activity, transferring phosphorus-containing groups	---	0.000000072
GO:0005198	F	structural molecule activity	---	0.00000053
GO:0003677	F	DNA binding	---	0.000001
GO:0016301	F	kinase activity	---	0.0000032
GO:0004518	F	nuclease activity	---	0.00034
GO:0003676	F	nucleic acid binding	---	0.0021
GO:0003774	F	motor activity	---	0.013
GO:0008135	F	translation factor activity, RNA binding	---	0.017
GO:0060089	F	molecular transducer activity	---	0.019
GO:0004872	F	receptor activity	---	0.019
GO:0003682	F	chromatin binding	---	0.021
GO:0004871	F	signal transducer activity	---	0.036
GO:0005623	C	cell	---	4.3E-187
GO:0044464	C	cell part	---	5.1E-187
GO:0005622	C	intracellular	---	1.7E-144
GO:0044424	C	intracellular part	---	1.8E-143
GO:0044422	C	organelle part	---	1.4E-128
GO:0044446	C	intracellular organelle part	---	1.8E-128
GO:0005737	C	cytoplasm	---	5.5E-111
GO:0043226	C	organelle	---	6.7E-111
GO:0043229	C	intracellular organelle	---	7.4E-111
GO:0043227	C	membrane-bounded organelle	---	8.9E-101
GO:0043231	C	intracellular membrane-bounded organelle	---	1.4E-100
GO:0044444	C	cytoplasmic part	---	4.8E-96
GO:0009536	C	plastid	---	3.1E-59
GO:0032991	C	macromolecular complex	---	3E-48
GO:0005829	C	cytosol	---	4.8E-47
GO:0031975	C	envelope	---	2.8E-32
GO:0031967	C	organelle envelope	---	2.8E-32
GO:0012505	C	endomembrane system	---	3.4E-28
GO:0043232	C	intracellular non-membrane-bounded organelle	---	7.6E-28
GO:0043228	C	non-membrane-bounded organelle	---	7.6E-28
GO:0005773	C	vacuole	---	6.1E-27
GO:0031974	C	membrane-enclosed lumen	---	5.2E-25
GO:0043233	C	organelle lumen	---	5.2E-25
GO:0070013	C	intracellular organelle lumen	---	5.2E-25
GO:0044428	C	nuclear part	---	6.7E-25
GO:0009579	C	thylakoid	---	2.5E-22
GO:0031981	C	nuclear lumen	---	1.1E-20
GO:0005634	C	nucleus	---	5.8E-19
GO:0005794	C	Golgi apparatus	---	1.7E-17
GO:0005783	C	endoplasmic reticulum	---	2.3E-13
GO:0005768	C	endosome	---	2.7E-11
GO:0005730	C	nucleolus	---	1.2E-10
GO:0030529	C	intracellular ribonucleoprotein complex	---	2.6E-10
GO:0005654	C	nucleoplasm	---	3.3E-09
GO:0005840	C	ribosome	---	0.000000049
GO:0005856	C	cytoskeleton	---	0.00000025
GO:0005777	C	peroxisome	---	0.00015
GO:0042579	C	microbody	---	0.00015
GO:0005635	C	nuclear envelope	---	0.00047
